# On the Ecology of Selenium Accumulation in Plants

**DOI:** 10.3390/plants8070197

**Published:** 2019-06-30

**Authors:** Elizabeth A. H. Pilon-Smits

**Affiliations:** Biology Department, Colorado State University, Fort Collins, CO 80523, USA; epsmits@colostate.edu; Tel.: +1-970-491-4991

**Keywords:** hyperaccumulation, plant symbiosis, plant adaptation

## Abstract

Plants accumulate and tolerate Se to varying degrees, up to 15,000 mg Se/kg dry weight for Se hyperaccumulators. Plant Se accumulation may exert positive or negative effects on other species in the community. The movement of plant Se into ecological partners may benefit them at low concentrations, but cause toxicity at high concentrations. Thus, Se accumulation can protect plants against Se-sensitive herbivores and pathogens (elemental defense) and reduce surrounding vegetation cover via high-Se litter deposition (elemental allelopathy). While hyperaccumulators negatively impact Se-sensitive ecological partners, they offer a niche for Se-tolerant partners, including beneficial microbial and pollinator symbionts as well as detrimental herbivores, pathogens, and competing plant species. These ecological effects of plant Se accumulation may facilitate the evolution of Se resistance in symbionts. Conversely, Se hyperaccumulation may evolve driven by increasing Se resistance in herbivores, pathogens, or plant neighbors; Se resistance also evolves in mutualist symbionts, minimizing the plant’s ecological cost. Interesting topics to address in future research are whether the ecological impacts of plant Se accumulation may affect species composition across trophic levels (favoring Se resistant taxa), and to what extent Se hyperaccumulators form a portal for Se into the local food chain and are important for Se cycling in the local ecosystem.

## 1. Two Faces of Se in Biology—An Introduction to Se Benefits and Toxicity

Mammals and many other animals, as well as many prokaryotes and some algae, require Se for their essential metabolism, as a structural component of selenoproteins [1], which have a variety of redox functions vital for immune, thyroid, and reproductive health [2]. In plants, essential Se metabolism appears to have been lost in evolution [1]. While not essential, Se can have physiological benefits to plants, which may be related to its tendency to upregulate plant antioxidant metabolites and enzymes, leading to a better capacity to scavenge reactive oxygen species (ROS) that impede plant performance, especially under stress [3]. Plants inadvertently take up and assimilate Se into organic forms due to the similarity of Se to the essential element sulfur (S), for which they have transporters and enzymes [4].

Selenium is toxic to organisms at elevated tissue levels, firstly because inorganic forms of Se can cause oxidative stress, and secondly because the organic selenocompounds selenocysteine (SeCys) and selenomethionine (SeMet) can get non-specifically incorporated into proteins, replacing their sulfur (S) analogues, which can compromise protein function [5]. For animals, long-term ingestion of food (e.g., plant material) with a Se concentration above 1 mg/kg DW can cause chronic Se toxicity (selenosis), and incidental ingestion of a high-Se meal (>1000 mg Se/kg DW) can cause acute toxicity, and even death [6]. The window between Se deficiency and Se toxicity in animals is extremely narrow: approximately one order of magnitude [2]. Natural soil Se levels vary dramatically worldwide. Seleniferous (Se-rich) soils, often derived from Cretaceous shale rock, are particularly common in North America, China, the Punjab area of India and Pakistan, and in Queensland, Australia. The variation in soil Se is associated with widespread problems with Se toxicity and (particularly) deficiency, estimated to affect over a billion people [7,8].

In plants, Se typically stimulates growth and stress resistance at 1–10 mg Se/kg DW [9], and the tissue concentration at which toxicity occurs is typically >100 mg Se/kg DW [10]. However, plants vary greatly in their Se accumulation and tolerance capacities. Some species called Se hyperaccumulators can tolerate tissue Se concentrations in the range of 1000–15,000 mg Se/kg DW and actively concentrate Se to these concentrations in all their organs while growing in their native seleniferous soils throughout the Western USA [11]. These hyperaccumulators have been reported in around fifty taxa from different families [12]. Apart from hyperaccumulators, a further distinction is made between non-accumulators, which contain less than 100 mg Se/kg DW, and Se accumulators, which contain 100–1000 mg Se/kg DW while growing on seleniferous soil [10]. Differences between plant species with respect to Se accumulation and tolerance may be due to differences in Se metabolism (forms of Se accumulated) and differences in tissue accumulation patterns. The next section will briefly review Se metabolism in plants.

## 2. What Can Plants Do with Se, at the Cellular and Whole-Plant Level?

All plants readily take up and accumulate Se in all their tissues, quite similar to their utilization of the analogous element S [4]. Selenate is the dominant bioavailable form of Se in soils, and thus the form of Se that typically enters the plant. It can be taken up and mobilized between plant organs via a family of sulfate transporter (SULTR) proteins [13]. The reductive sulfate assimilation pathway can metabolize selenate to selenocysteine (SeCys), and the resulting SeCys may get incorporated into protein (potentially toxic), or may be further metabolized via selenomethionine (SeMet) to volatile dimethylselenide (DMSe) [4,10,14]. In hyperaccumulator species, most Se is found as methyl-SeCys, due to a high SeCys methylation activity, which is considered a Se tolerance mechanism [15]. Methyl-SeCys may be further converted to volatile dimethyldiselenide (DMDSe) [10,14]. Non-hyperaccumulator species tend to have a slower rate of selenate assimilation than hyperaccumulators, and therefore accumulate relatively more inorganic Se [16,17,18]. Other differences between hyperaccumulators and non-hyperaccumulators are that the former translocate Se more in their xylem (from root to shoot) and phloem (from leaves to reproductive organs), and often sequester Se in specialized tissues such as leaf hairs or epidermis [19,20,21]. Furthermore, hyperaccumulators appear to enrich themselves with Se over S compared to their growth substrate or neighboring vegetation, perhaps owing to an ability to discriminate between the two elements [22,23,24].

**Figure 1:**
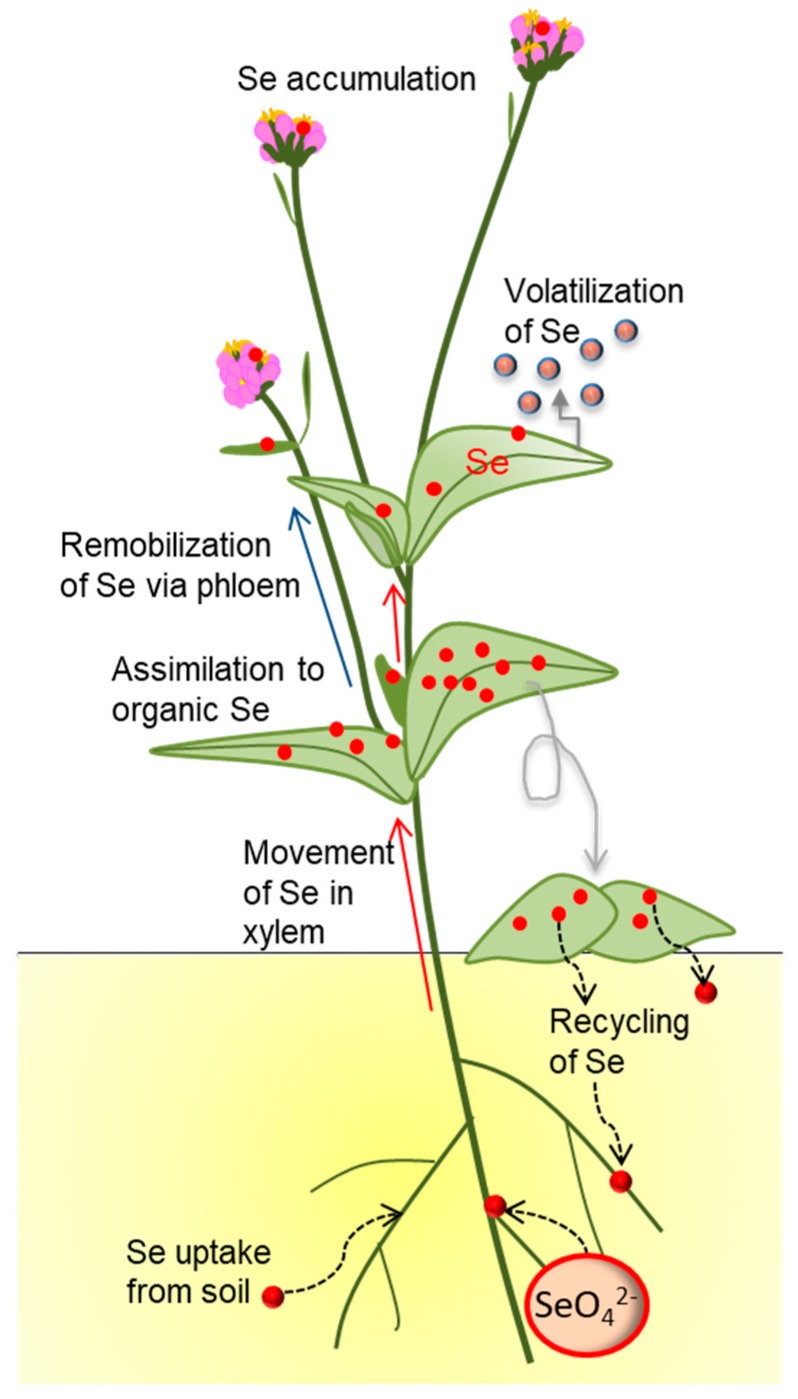
Accumulation and transformation of Se (red circles) by plants, and effects on local Se cycling.

When plants grow in a Se-containing environment, they bioconcentrate the locally available Se in their tissues and, depending on the species, also biotransform the inorganic Se to organic Se to a varying degree (Figure 1). This accumulated, organic/inorganic plant Se is recycled when the plants shed leaves or die, or in root exudation or through volatilization. When the plant is consumed, the Se may also move into higher trophic levels. The concentrations and forms of Se in the different plant parts matter for these Se fluxes, because they differ in nutritional value and toxicity. Considering the dual beneficial and toxic effects of Se on organisms, it is likely that Se accumulation in different plant parts in different forms will affect ecological partners. These collective ecological effects associated with plant Se accumulation will be reviewed and discussed below.

## 3. Ecological Effects of Plant-Accumulated Se on Ecological Partners

### 3.1. Plant–Herbivore Interactions

There is broad evidence [25] from field and laboratory studies that Se in plant tissues, as well as volatile Se emitted by plants, deters a variety of generalist herbivores, including invertebrates with different feeding modes (phloem feeders, leaf chewers, cell disrupters) [26,27,28,29,30,31,32] and vertebrates [33]; moreover, Se in plant tissues is toxic to generalist herbivores. Both the Se in hyperaccumulators (often in organic forms) and in non-hyperaccumulators (often inorganic Se) have these protective effects. The degree of protection increases with Se concentration, because some herbivores are more Se sensitive (e.g., aphids, [29]) while others can resist a higher Se concentration (e.g., spider mites [32]). The protective effect appears to come from the Se directly, because adding Se to insect food at levels relevant for those accumulated in plants also causes toxicity [26,27]. However, it cannot be excluded that Se additionally upregulates the production of other defense compounds. The interaction between Se and (other) defense compounds is an area that is relatively under-studied [34].

Most studies on the effects of Se on plant–herbivore interactions were carried out in a controlled laboratory setting, in the form of choice and non-choice feeding experiments. In addition, a few field manipulative studies were carried out in which Se hyperaccumulator *Stanleya pinnata* (Brassicaceae) pretreated with or without Se were planted in a naturally seleniferous habitat in Colorado, USA, and herbivory and survival were scored. The plants pretreated with Se (containing 50 mg Se/kg DW) survived better and suffered less herbivory, both in the course of several months when grasshoppers were the predominant herbivore [30], as well as over a period of two years when prairie dogs were the main herbivore [33].

The finding that Se at low concentrations (< 10 mg/kg DW) is already protective against herbivory and that the protective effect is correlated with Se concentration provides insight into the evolution of Se (hyper)accumulators from nonaccumulators, and the likely selection pressures that continuously favor increasing Se levels [25]. At some point during the evolution of Se hyperaccumulation, the plant must have evolved unique Se tolerance mechanisms (conversion to methyl-SeCys, tissue-specific sequestration) to withstand the ever increasing Se levels favored by herbivory selection pressure.

The finding that Se-containing plants deter generalist herbivores and are toxic when consumed also offers insight into the potential ecological effects of high-Se plants in the field, either in natural settings or when cultivated on seleniferous soils. In agricultural settings, the protective effects of Se likely will reduce the need for pesticide application. In natural settings, the high-Se plants may be expected to affect local herbivore abundance and composition and may change herbivore load on local nonaccumulator plant species. On the one hand, the volatile Se produced by high-Se plants may deter herbivores across a certain area of influence that may also benefit neighbors, but on the other hand, the herbivores deterred from high-Se plants may pose extra herbivory pressure on low-Se neighbors. A field survey comparing invertebrate load and composition between two Se hyperaccumulator and two non-accumulator species growing in the same seleniferous area found overall lower herbivore load on the hyperaccumulators as well as lower and altered species diversity [35]. This finding suggests that the Se in hyperaccumulator plants lowers their susceptibility to herbivory in their natural habitat, but that some herbivores are able to use hyperaccumulators as a food source. Indeed, several studies provide evidence that Se hyperaccumulator plants harbor Se resistant herbivores [25]. Seeds of the hyperaccumulators *S. pinnata* and *Astragalus bisulcatus* (Fabaceae) collected in their native seleniferous habitat both were found to harbor different beetle, wasp, or chalcid herbivores [36]. While the seeds contained 5000–10,000 mg Se/kg DW, the seed herbivores emerging from these seeds contained only ~10 mg/kg DW, so their Se resistance mechanism is exclusion. For clarification: the term resistance is used here to indicate the ability to withstand high external Se levels, while the term tolerance is used to indicate the ability to withstand high internal (tissue) Se concentration. Leaves of these same two hyperaccumulator species were found to harbor different species of moths, which were both preyed on by their own species of parasitic wasp [37,38]. The leaves contained 2000–10,000 mg Se/kg DW and the herbivores around 250 mg Se/kg DW; this points to a certain degree of Se exclusion, but also to Se tolerance, considering that other Lepidoptera larvae suffered mortality at tissue Se concentrations around 25 mg/kg DW [28,37]. Based on comparison of the diamondback moth *(Plutella xylostella*) variety found on *S. pinnata* in its natural seleniferous habitat in Colorado (USA) with a diamondback moth variety from a low-Se area, the CO diamondback moth appears to have lost its aversion to feed or oviposit on high-Se plants, as well as its capacity to break down methyl-SeCys to more toxic forms of Se [37]. Interestingly, the diamondback moth is a recent invasive species in Colorado, and notorious for its ability to overcome plant defenses and manmade pesticides. Overcoming the elemental defense of Se hyperaccumulators fits into this pattern.

**Figure 2:**
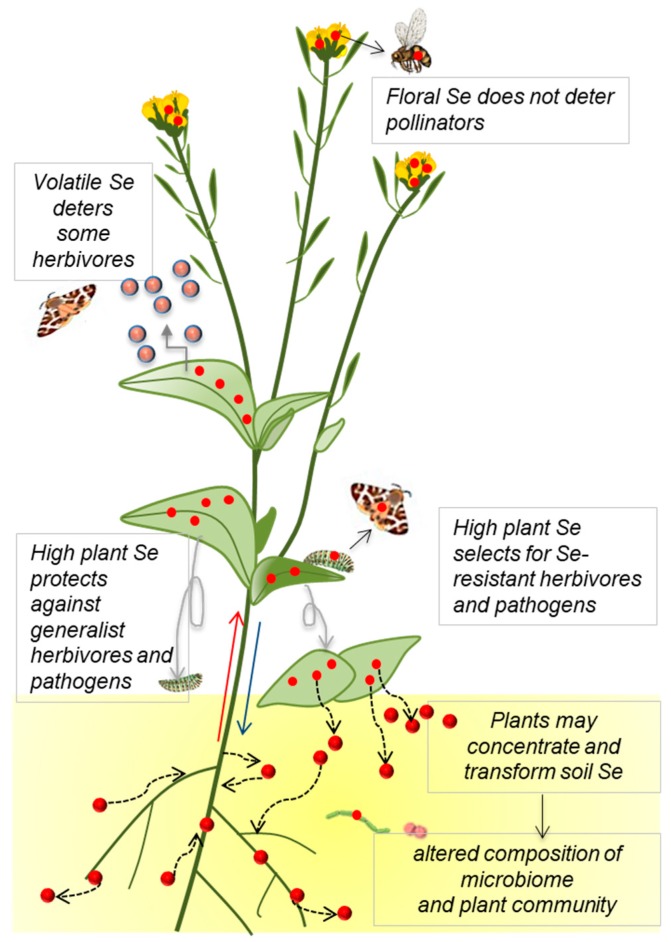
Effects of plant Se accumulation on local soil Se (red circles) distribution and on ecological interactions, and the implications for Se movement into the food chain. Accumulation and transformation of Se (represented by red circles) by plants, and effects on local Se cycling.

In summary, these collective plant–herbivore interaction studies indicate that Se accumulation in plants negatively affects generalist herbivores (via deterrence and toxicity), thereby protecting the plants, but also favoring the evolution of Se resistance in herbivores, which as a result can exclusively utilize high-Se plants as a food source (Figure 2). Potential areas for further investigation are whether any of these Se-resistant herbivores are specialists, and whether plant-derived Se may protect Se-tolerant herbivores from predators. The finding that arthropod composition was altered on hyperaccumulators and that some herbivores contain fairly high Se levels and are parasitized by predators that also contain high levels of Se suggests that Se hyperaccumulators may form a portal for Se into the local food chain, and may play a role in local Se cycling. The extent to which this is a significant factor is a question that remains to be investigated.

### 3.2. Plant–Pollinator Interactions

Plants accumulate Se in all their organs, including flowers. Hyperaccumulators even contain the highest Se concentrations in their flowers and fruits, including the pollen, nectar, and seeds [20]. Considering the observation that high-Se material deters invertebrate herbivores and is toxic to them when ingested, it may be expected that high-Se floral parts also deter invertebrate pollinators. However, no evidence has been found that this is the case. Selenium-treated hyperaccumulator (*S. pinnata*) and non-hyperaccumulator (*Brassica juncea*, Brassicaceae) plants with leaf and floral Se concentrations up to 4000 mg/kg DW were visited to equal extent as their non-Se treated counterparts by potential pollinators in the field in Colorado, which included both native bumble bees and non-native honey bees [20]. Similarly, in a separate study with honey bees, no evidence was found for a deterrent effect of Se on honey bee feeding [39]. Bumble bees and honey bees collected in the field from Se hyperaccumulators were found to contain Se in their tissues as well as in their pollen baskets, so appear to actively forage the high-Se pollen and nectar and likely feed it to their brood [20]. Therefore, the high Se in the floral parts of plants does not appear to impair plant reproductive fitness through negative effects on pollinator visitation, which is relevant when considering the evolutionary benefits and costs of hyperaccumulation, and also when considering the effects of accumulated Se on the local ecology in an agricultural setting, such as during Se phytoremediation.

An important question that still awaits further study is whether and how the collected plant Se affects the health of the pollinators. From feeding studies with Se-spiked sugar water, it is clear that inorganic and organic Se are toxic to honey bees at the levels found in high-Se plants [39]. There may be a difference in this respect between non-native honey bees and native bumble bees, considering the finding that bumble bees collected from hyperaccumulators in a seleniferous habitat in CO contained, on average, 250 mg Se/kg DW in the form of methyl-SeCys, while honey bees contained only 15 mg Se/kg DW in a variety of inorganic and organic chemical forms [20]. This may reflect differences in foraging behavior, but may also reflect differences in Se tolerance. The Se levels found in the native bumble bee are similar to those found in Se-tolerant herbivores found in the same area, while the Se levels found in the non-native honey bee are similar to the Se levels found to be lethal to Se-sensitive Lepidoptera larvae [37]. It is possible that Se hyperaccumulator pollination services are carried out by special Se-tolerant pollinators that can utilize the hyperaccumulator as a food source, and that may even benefit from their elevated Se levels through reduced predation.

In summary, plant–pollinator interaction studies so far indicate that Se accumulation in plants does not deter pollinators and does not affect pollinator visitation (Figure 2). Whether plant-derived Se positively or negatively affects pollinators remains to be determined and is an important question to address. In seleniferous areas, Se hyperaccumulators may favor the evolution of Se tolerant pollinators that service and utilize hyperaccumulators without ill effects. In analogy with Se-tolerant herbivores, Se-tolerant pollinators may be specialists and may be protected by their accumulated Se from predators, and they form another pathway for plant-accumulated Se into the food chain. These questions await further study.

### 3.3. Plant–Microbe Interactions

The plant microbiome consists of prokaryotic and fungal symbionts that live inside the plant (endosphere), or on or near the plant’s shoot or root surface (phyllosphere, rhizosphere), where they benefit from the plant’s release of organic carbon compounds [40]. Many of these microbes benefit the plant by helping to acquire water and nutrients, stimulating plant growth or innate immunity, producing ecologically significant compounds, or fighting off pathogens [41]. However, some microbes are pathogens, and negatively affect their host. High Se levels in different plant parts may be expected to affect the associated microbiome’s abundance or composition by negatively affecting Se-sensitive taxa, perhaps positively affecting others that can use Se metabolically, or just by altering the competitive interactions. If plant Se would negatively affect microbiome abundance, this may have a fitness cost for the plant because of loss of beneficial interactions. On the other hand, if plant Se would be toxic to pathogens, this may offer the plant a fitness benefit. Several studies have studied these effects.

Hanson and coworkers [28] showed that Se treatment could protect *B. juncea* from two fungal pathogens, one a leaf fungus (*Alternaria brassicicola*) and the other a stem/root fungus (*Fusarium* sp.). Indeed, these fungi showed sensitivity to Se levels like those found in the plants when grown on medium spiked with Se. Thus, Se may protect plants from generalist Se-sensitive fungal pathogens. However, there are leaf fungal pathogens in seleniferous areas that can utilize Se hyperaccumulators as a host: an *Albugo* species is regularly observed on leaves of wild *S. pinnata* containing 2000 mg Se/kg DW in Colorado, USA (Ami Wangeline and Elizabeth Pilon-Smits, unpublished results).

A study by Wangeline and coworkers [42] found that rhizosphere fungi from plants growing in seleniferous areas had overall higher Se resistance than rhizosphere fungi isolated from plants growing in a lower Se area. There was no difference in Se resistance between rhizosphere fungi isolated from Se hyperaccumulators and non-accumulators, but it should be noted that the Se resistance was tested at a fairly low concentration. It was hypothesized that the higher overall Se levels in the seleniferous area may have a selective effect on the fungal community, leading to higher Se resistance. Some of the fungal isolates from roots of hyperaccumulators were also found to occur as seed and stem endophytes [38]. In plant inoculation studies, several of the fungal isolates found to influence plant Se accumulation and growth, both of their hyperaccumulator host and of related non-hyperaccumulators [43,44]. Certain mycorrhizal fungal isolates have also been shown to be able to affect plant growth and Se accumulation [45,46,47]. Endophytic microbes may also influence Se speciation in hyperaccumulators to a degree, as suggested by the presence of elemental Se (Se0) in microbe-colonized plants; many microbes convert selenate or selenite to Se0 as a Se tolerance mechanism. Hyperaccumulators *A. bisulcatus* and *S. pinnata* contained up to 30% elemental Se (Se0) in their natural habitat, but only accumulated organic Se when grown from surface-sterilized seed in a greenhouse [48]. Similarly, up to 30% of the Se in *A. bisulcatus* root nodules harboring a N_2_ fixing bacterial endosymbiont was Se0, while the adjacent roots accumulated only organic Se [38]. The interactions of plant Se and nodulation were investigated by Alford and coworkers [17,49], who found that nodulated plants accumulated more Se, particularly in the form of gamma-glutamyl-methyl-SeCys. It was hypothesized that the bacterial symbiont may play a significant role in providing the nitrogen for this compound, particularly for the glutamyl-moiety, since the concentration of methyl-SeCys was not affected by nodulation. A field survey of six Se hyperaccumulator and non-accumulator *Astragalus* species showed no significant differences in nodulation between the groups. Thus, there is no evidence for a cost of Se hyperaccumulation in *Astragalus* through negative effects on nitrogen fixing endosymbionts. It is possible that hyperaccumulator-associated Rhizobia have evolved enhanced Se resistance; their isolation and characterization will be interesting to pursue in future studies.

Sura-de Jong et al. [50] and Cochran et al. [51] studied the prokaryotic microbiomes of the endosphere and rhizosphere, respectively, of different plant species growing in a seleniferous area in Colorado, USA. There was no difference in Se resistance between isolates from hyperaccumulator and non-hyperaccumulator species. Interestingly, most of the isolates were highly Se resistant: most were unaffected up to 10 mM selenate or selenite, and many could withstand 200 mM of both compounds. All were able to reduce selenite to Se0, a likely tolerance mechanism. Terminal restriction fragment length polymorphism (T-RFLP) analysis found no effect of Se hyperaccumulation on endophytic species diversity [50]. Illumina16S rRNA sequencing revealed that the rhizosphere microbiomes of hyperaccumulators were more similar to each other than to those of non-hyperaccumulator relatives on the same site, and showed higher species richness [51].

Prokaryotic isolates from the endosphere and rhizosphere of hyperaccumulators, or from various other environmental sources have also been studied for their effects on plant growth and Se accumulation. Enhanced plant growth and Se accumulation was found after bacterial inoculation in many different studies using host and non-host species, hyperaccumulators, and non-hyperaccumulators, and wild and crop species [50,52,53,54,55,56,57]. In addition to Se accumulation, Se volatilization could be enhanced in several terrestrial and aquatic species by inoculation with bacteria isolated from a Se-polluted area [52,53].

In summary, there is no evidence that high plant Se levels have an evolutionary cost by negatively affecting the plant’s beneficial symbiotic interactions with fungi or prokaryotes. However, there is evidence that Se can protect plants from certain pathogenic fungi. Prokaryotes generally appear to be highly Se resistant, and thus likely will not suffer toxicity at the Se levels found in plants. Nevertheless, Se accumulation by plants appears to affect the composition of the plant microbiome. While this likely does not reflect differences in Se tolerance, it is possible that some microbes can benefit more from the plant-associated Se than other ones. Thus, Se may affect competitive interactions and microbial fitness. Microbial species richness may also be positively affected by plant Se accumulation, because it creates more variation (heterogeneity) and thus more niches to occupy. There is ample evidence that fungi and bacteria can positively affect plant growth and Se accumulation and volatilization, and these effects are not restricted to their natural host. This may have applications in Se biofortification (production of nutritionally enhanced crops) and phytoremediation (plant-based environmental cleanup) [41,58]. This is a promising research area and is increasingly attracting attention from agronomists and farmers.

### 3.4. Plant–Plant Interactions

There is evidence that high-Se plants can enhance the tissue Se levels in neighboring plants in seleniferous natural areas: different individuals of the same species showed up to 20-fold higher tissue Se concentrations when they were growing in close proximity to hyperaccumulators [59,60]. The underlying mechanism may be that the large, perennial hyperaccumulator plants scavenge a large soil volume for Se, accumulate it in their tissues, convert it to organic forms, and then release these highly bioavailable selenocompounds in their surroundings via leaf litter, root turnover and exudation, and perhaps volatilization. Indeed, the soil under the canopy of hyperaccumulators was found to be enriched in Se by 7–11 fold [59]. Of course, whether these Se “hot spots” around hyperaccumulators are caused by the plant, or rather preceded the colonization by the hyperaccumulator and facilitated their establishment, is hard to determine, but several indications point to the plant as the cause rather than the result of the Se concentration spots. Litterbag decomposition studies of hyperaccumulator and non-accumulator litter showed that the high-Se litter bags lost most of their Se within 6–12 months and enriched the soil below the litter bag with Se [61]. The form of Se in the soil surrounding hyperaccumulators was predominantly organic, in the same form as that found in all the hyperaccumulator organs, as well as in root exudate [62]. Furthermore, paired surveys of hyperaccumulators and nearby non-accumulator control plants showed significantly higher soil Se levels around the hyperaccumulators, and the soil Se concentrations decreased rapidly beyond the canopy of the hyperaccumulators (Jason Reynolds and Elizabeth Pilon-Smits, unpublished results).

**Figure 3:**
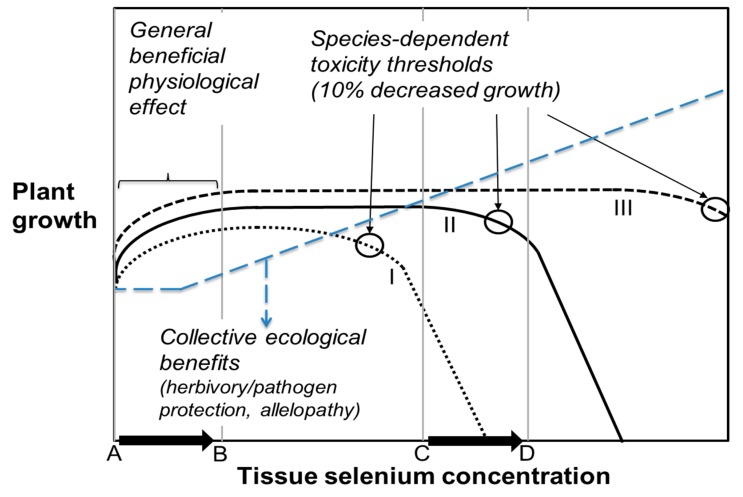
Beneficial and toxic effects of Se on plants. Black lines show growth for species that are Se sensitive non-accumulators (I), moderately tolerant Se accumulators (II), or highly tolerant Se hyperaccumulators (III). All benefit from Se at low tissue levels (A→B), but their toxicity thresholds vary greatly, from <100 mg/kg DW to >15,000 mg/kg DW. Increased tissue Se concentration (C→D), e.g., due to ecological interactions with other plants, may lead to either death (I), decreased growth (II), or continued physiological benefit (III). The diagonal blue line (large dash) indicates increasing ecological benefits of tissue Se accumulation due to protection from herbivory and fungal pathogen infection.

The high-Se soil surrounding hyperaccumulators and concomitant increase in tissue Se concentration in neighboring plants may affect these neighbors differently, depending on their Se tolerance. To Se-sensitive plant species (Figure 3, species I), the soil surrounding hyperaccumulators can easily become toxic, leading to reduced germination and growth [59]. However, Se-tolerant species (Figure 3, species III) may actually benefit from their enhanced Se levels when growing next to Se hyperaccumulators, owing to herbivory protection and/or physiological benefits [57,60]. For species with intermediate Se tolerance (Figure 3, species II), the effects may be positive or negative, depending on the tissue Se concentration (Figure 3, A→B vs. C→D).

If Se hyperaccumulators have a negative effect on surrounding Se-sensitive vegetation and a positive effect on Se-tolerant vegetation, it is possible that over time the plant species composition is influenced toward relatively more Se-tolerant Se accumulating species and individuals. If that were the case, it would likely also affect higher trophic levels and microbial composition, and perhaps Se cycling in the local ecosystem. This is an interesting area for further research. From an applied perspective, it may be interesting to co-cultivate or alternate Se hyperaccumulators with regular crop species on naturally seleniferous or Se-contaminated soils, or to use hyperaccumulator material, such as green manure with which to fertilize crops. This may enhance Se accumulation in the crop species, and alter the crop’s Se speciation toward more organic (nutritious) forms. These practices may benefit Se phytoremediation, but also may create Se biofortified plant products that may help alleviate Se deficiency in humans or livestock elsewhere.

## 4. Integrative Discussion on the Ecological Implications of Plant Se Accumulation

The aim of this section is to summarize and interpret the various ecological implications that Se (hyper)accumulation may have on the plant itself as well as the surrounding local ecosystem. There are some general trends emerging from the various studies on the ecological effects of plant Se accumulation, which were already noted in our earlier review in 2012 [25], and have been supported by follow-up studies. Additionally, recent studies have given better insight into larger scale effects of the presence of high-Se plants on the overall composition of associated microbiomes and plant communities. A universal pattern emerges from the findings so far (summarized in Table 1): whether the interactions involve microbes, herbivores, pollinators, or other plants, the trend appears to be that the high Se concentrations in and around hyperaccumulators negatively affect Se-sensitive partners but also offers a niche for Se-tolerant specialist partners. The negative effects on generalist herbivores and Se-sensitive plants benefit the plants via reduced herbivory [35], and lower abundance of surrounding vegetation [59]. The presence of the high Se levels in and around hyperaccumulators may have acted as a selection pressure for the evolution of Se tolerant herbivores, pollinators, detrivores, fungi, and neighboring plants. Conversely, the evolution of Se-tolerant herbivores and pathogens may have contributed to the evolution of Se hyperaccumulation to ever-increasing concentrations. Because of the existence of Se-tolerant mutualistic partners, Se hyperaccumulation may not carry an evolutionary cost in the form of detrimental effects on microbial partnerships or insect pollination.

The presence at different trophic levels of Se-tolerant species that can accumulate Se to substantial concentrations likely contributes to the movement of Se in the local food chain, and ultimately to Se cycling in the local ecosystem. The Se hyperaccumulator plants form the entry point into this movement, and may be an important factor. At this point, not very much is known about the relative contribution of terrestrial vegetation in global Se cycling [8]. This should be an interesting topic of further study.

## Figures and Tables

**Table 1:** Overview of ecological aspects of plant Se accumulation, as observed on hyperaccumulators (HA) and/or non-HA in laboratory (L) and/or field (F). References for the summarized observations can be found in the text, at the indicated sections, where more background information is provided.

**Plant-herbivore interactions (Section 3.1)**		
*Generalist herbivores (Se-sensitive)*		
Plant Se deters herbivores from feeding (many invertebrate taxa, prairie dogs)	HA, non-HA	L, F
Plant Se deters herbivores from ovipositing (moth)	HA	L
Plant Se is toxic, even deadly to herbivores when ingested (many invert taxa)	HA, non-HA	L
High-Se plants harbor fewer invertebrates in the field	HA	F
*Potential specialist herbivores (Se-resistant)*		
High-Se plants harbor Se-excluding seed herbivores (bruchid, chalcid inverts)	HA	F
High-Se plants harbor leaf herbivores, some proven Se-tolerant (invert taxa)	HA	L, F
High-Se litter harbors higher levels of micro-arthropod decomposers than low-Se litter, and decomposes faster	HA, non-HA	F
*Selenium movement from herbivores to higher trophic levels*		
Se-tolerant herbivores are parasitized by Se-tolerant wasps (2 moths, 2 wasps)	HA	F
High-Se plants harbor predator inverts with elevated Se levels (many taxa)	HA	F
**Plant-pollinator interactions (Section 3.2)**		
*Generalist pollinators (Se-sensitive)*		
High-Se flowers or feeding solution do not deter foraging by invert pollinators	HA, non-HA	L, F
High-Se pollen and nectar is collected and ingested by honey bees	HA	F
High-Se food sources (chemical) are toxic to honey bees	non-plant	L
*Potential specialist pollinators (Se-tolerant)*		
Native pollinators accumulate Se to high levels from high-Se flowers	HA, non-HA	F
**Plant-microbe interactions (Section 3.3)**		
*Generalist microbes (default for fungi: Se-sensitive; for prokaryotes: Se resistant)*		
High-Se plants are protected from pathogenic fungi (2 species)	non-HA	L
Fungi from seleniferous/non-seleniferous soils differ in Se resistance	HA, non-HA	F, L
Se level in host plant is not correlated with Se resistance in fungal symbionts	HA, non-HA	F, L
Se level in host plant is not correlated with Se resistance in bacterial symbionts	HA, non-HA	F, L
Se level in host plant affects rhizosphere microbiome composition, (+) sp. richness	HA, non-HA	F
Microbes (isolated strains or consortia) can affect plant Se accumulation, volatilization, metabolism, tolerance, and general plant growth	HA, non-HA	L
*Potential specialist microbes*		
High-Se plants are observed to harbor leaf fungus (1 species)	HA	F
High-Se litter harbors higher levels of cultivable microbes than low-Se litter, and decomposes faster	HA, non-HA	F
Rhizobial endosymbiont in root nodules may affect Se accumulation and Se metabolism in host plant	HA	F, L
Fungal endosymbionts may affect Se accumulation and Se metabolism in host	HA	F, L
Certain bacterial taxa are over-abundant in rhizosphere of Se HA	HA, non-HA	F
**Plant-plant interactions (Section 3.4)**		
*General effects*		
Soil around HA plants in field is ~10 fold elevated in Se, mostly in organic form	HA, non-HA	F
Soil around high-Se plants in field inhibits germination, growth of *Arabidopsis*	HA	F, L
Vegetative cover is reduced around HA plants in the field, and species composition is different	HA, non-HA	F
*Potential specialist plant species*		
Some Se-tolerant plant species co-occur with HAs in field; they accumulate more Se, have less herbivory and grow better because of this association	HA	F
